# PSMA-based theranostics in diagnosing and treating prostate cancer in the Asian male population: a narrative review

**DOI:** 10.3389/fonc.2025.1655082

**Published:** 2025-09-05

**Authors:** Feng Liu, Chang Ge, Bingzhang Qiao, Zubeila Aihemaiti, Zhao Li, Weijie Zhang, Abudureheman Zebibula, Mulati Rexiati

**Affiliations:** ^1^ Department of Urology, The Central Hospital of Shaoyang, Shaoyang, Hunan, China; ^2^ Department of Urology, The First Affiliated Hospital of Xinjiang Medical University, Urumqi, Xinjiang, China; ^3^ Key Laboratory of Genitourinary Diseases, Xinjiang Medical University, Urumqi, Xinjiang, China

**Keywords:** prostate cancer, prostate-specific membrane antigen, positron emission tomography, theranostics, radioligand therapy

## Abstract

Prostate-specific membrane antigen (PSMA) is a protein primarily overexpressed on the surface of prostate cancer (PCa) cells, making it a key target for PSMA-based theranostics, which combine diagnostic imaging and therapy. PSMA-based molecular probes, conjugated tracers and isotopes, and multifunctional imaging technologies have significantly advanced the landscape of high-risk PCa management, particularly during initial diagnosis and treatment planning. This tool is especially crucial as the ratio of mortality to incidence of PCa in Asian populations is higher, and the overall prognosis is significantly worse compared to Western countries. Furthermore, prostate-specific antigen (PSA) screening using multiparametric magnetic resonance imaging (MRI) and pathological examination shows that only a small percentage of men (below 30%) with PSA levels between 4–10 ng/ml in China, considered low risk, actually test positive for PCa when biopsied. Therefore, PSMA ligand-based positron emission tomography (PET) has been increasingly utilized for the accurate diagnosis, clinical staging, dynamic monitoring, treatment guidance, and prognosis evaluation of PCa. Moreover, PSMA-targeted radioligand therapy (RLT), antibody-drug conjugate (ADC) therapy, cellular immunotherapy, photodynamic therapy (PDT), and photothermal therapy (PTT), along with PSMA radioguided surgery (PSMA-RGS) intervention, have shown substantial advantages and promising potential. The field of PSMA ligands in PCa management has seen remarkable advancements in recent years, impacting both diagnostic and therapeutic approaches. This review discusses and summarizes the recent research progress and application prospects of PSMA-based theranostics in the clinical management of PCa in Asian populations.

## 1 Introduction

Recent years have shown a continuous increase in the incidence of prostate cancer (PCa), especially in Europe, North America, and Oceania (1). An analysis of global trends in PCa indicates worsening health disparities between more developed [higher Social Development Index (SDI)] and less developed nations, with PCa burden showing a substantial increase in the Slope Index of Inequality (SII) from 329.90 in 1990 to 544.03 in 2021 (2). Although the Concentration Index (CI) shows a reduction in the concentration of PCa burden in SDI countries (from 0.44 in 1990 to 0.31 in 2021), the burden of PCa is more concentrated in these countries compared to low SDI countries. This can be attributed to healthcare access and screening facilities, and education about PCa. Furthermore, age-standardized rates (ASR) for PCa increased significantly worldwide, and the incidence rate is expected to increase notably in China between 2022 and 2046. With the population aged ≥65 years expected to reach 1.6 billion, age may become a primary determinant of PCa incidence in the future (3). These findings align with the rapidly aging population in Asia, which is expected to contribute to a continued rise in incidence and prevalence of PCa among Asian men (4). Historically, the incidence rate of PCa in Asian men has been observed to be lower compared to Western countries; a study by Siegel et al. (5) showed that Black men had a PCa incidence 1.3 times that of white men, and Asian men had a PCa incidence 0.7 times that of White men. Moreover, Down et al. (6) also came to a similar conclusion, and found that Black men had the highest PCa incidence at 24.7% (95% CI 23.3%, 26.2%); Asian men had the lowest at 13.4% (95% CI 12.2%, 14.7%); and the incidence for White men was 19.8% (95% CI 19.4%, 20.2%). However, the mortality-to-incidence ratio (MR/IR) of Asian men is higher, the 5-year survival rate is lower, and patients present with advanced-stage and metastatic disease (7). In 2011, a study showed that the MR/IR in Asia ranged from 0.3 to 0.6, whereas it was 0.12 in North America and 0.20 in Europe (8). The MR/IR (0.44) was significantly higher in Asian countries than in other places, except for Africa, suggesting that PCa poses a particularly significant health threat to the Asian population. In a study conducted by Zhang et al. (9), they discovered that PCa in Chinese and US populations exhibits notable differences in clinicopathologic features. Chinese patients tend to be older and harbor a higher proportion of poorly differentiated tumors with more advanced grade groups (Groups 4 and 5 were observed in 25% of Chinese patients compared to 17.11% of the USA cohort). In many Asian countries, PCa is frequently diagnosed at later stages, often after it has metastasized. This stands in stark contrast to countries like the United States, where a majority of PCa cases are diagnosed at an early, localized stage. Studies from China, for instance, reveal that over 60% of PCa patients are diagnosed at advanced stages, compared to the US, where 70% are diagnosed early (7). Additionally, the age-standardized 5-year overall survival rate for Chinese PCa patients (69.2%) is significantly lower than that observed in the United States (97.4%). These issues may be a result of late-stage diagnosis, limited access to prostate-specific antigen (PSA) screening, and less sensitive tests for Asian men (4, 10).

Furthermore, the PCa composition and characteristics in Asian men are different from other regions. Findings from a tumor marker analysis have identified distinct genetic variants and different frequencies of risk alleles for PCa in Asian men compared to Western populations, with a higher proportion of high-risk cases and different clinical and genomic characteristics (11). Studies suggest that specific gene mutations and variations linked to PCa are observed at different frequencies across racial groups (12). For example, studies have indicated that PTEN loss and TMPRSS2-ERG fusion are more prevalent in White and Black men than in Asian men. TMPRSS2-ERG fusion was discovered to have a prevalence of 50% in White men, but lower frequencies were reported in Asian populations (8-21%) (13). PTEN inactivation was reported in 70% of White men, and only 34% in Chinese patients. Again, current clinical tests cannot detect PCa patients with highly metastatic prostate cancer (mPCa), which accounts for about 30% of all newly diagnosed PCa in Central Asia (14). Consequently, advancements in diagnostic and therapeutic technologies and continued research into novel biomarkers are needed to offer promising avenues for improved detection and management of PCa.

PSA screening is a widely used PCa diagnosis biomarker currently recommended by international guidelines. Despite its wide application, PSA has limitations, particularly in the gray zone (4–10 ng/ml), with biopsy rates of 25% in China and 40% in America. Even with PSA levels over 20 ng/ml, the positive biopsy rate was reported as only 70% (10). Furthermore, in a landmark randomized controlled trial that recruited 61,000 men, approximately 76% of the biopsies performed for an elevated PSA level were false positives, illustrating PSA screening’s low specificity (15). This limitation can lead to increased prostate biopsies, which carry potential risks of infection ranging from 0.5% to 10.1% (16, 17). These findings show that PSA is an organ-specific marker but not disease-specific, and its elevation can be caused by factors other than PCa, such as non-cancerous inflammation and benign prostatic hyperplasia (4). This is an issue particularly in Asian men, as there is a higher rate of false positives in Asian populations compared to Western populations (6). Furthermore, some studies suggest that Asian men may have lower PSA levels overall, which could affect the accuracy of standard PSA cutoffs (4). Asian men in the UK were reported to have lower PSA levels at diagnosis compared to white men (18). Liu et al. (19) also found that serum PSA values in Chinese men older than 50 years were lower than those in other races, making the optimal PSA cutoff for PCa detection in the Chinese population unclear. Therefore, in PSA testing, it is necessary to fully consider racial differences and develop targeted screening and diagnostic strategies to improve outcomes and reduce overdiagnosis.

**Figure 1:**
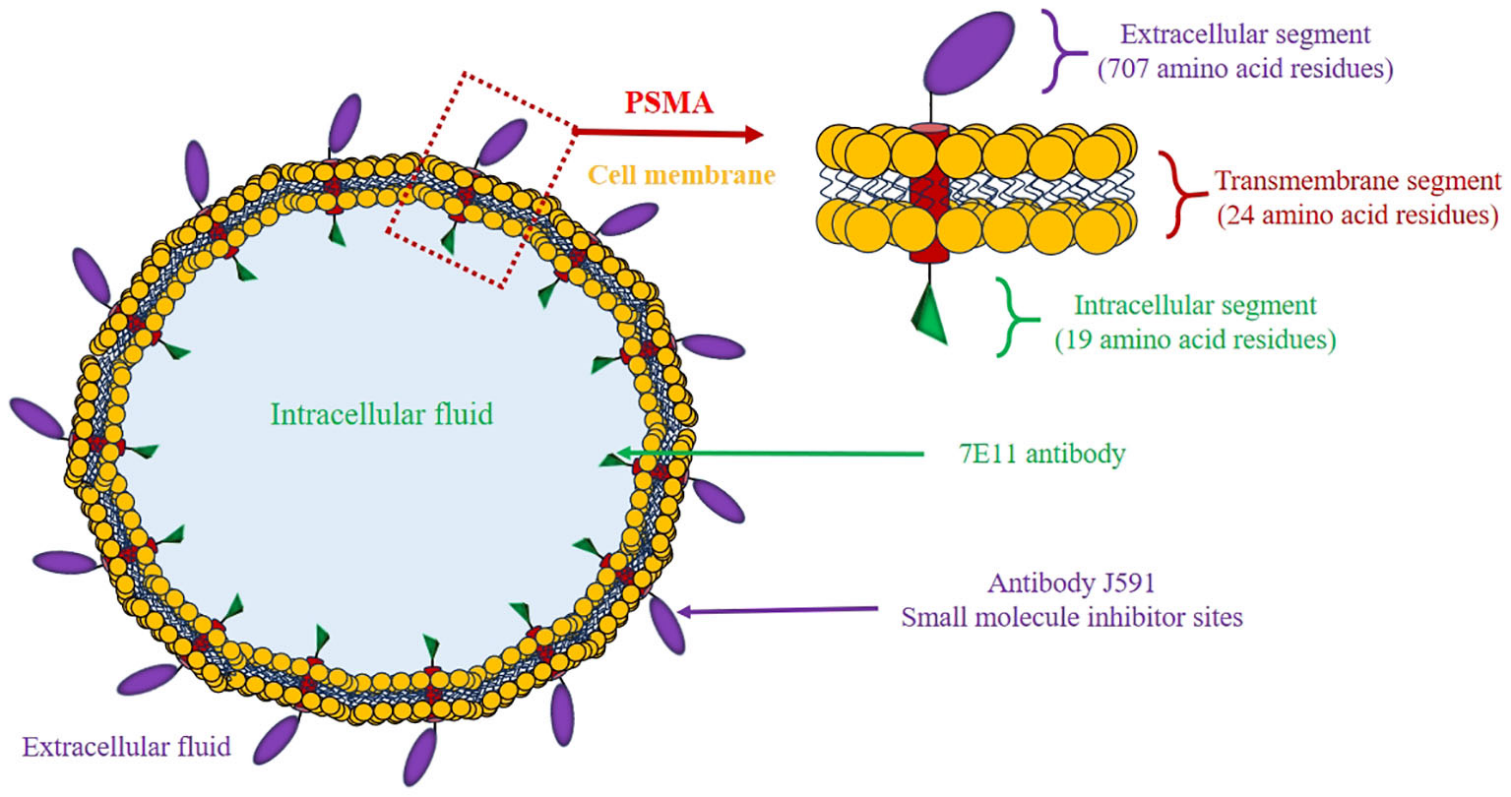
Schematic illustration of the PSMA structure.

Prostate-specific membrane antigen (PSMA) is a type II transmembrane glycoprotein (20) (**Figure 1**) commonly found to be highly expressed in prostate tumor cells, and its levels are about 100–1000 times higher than in normal prostate tissues and other tissues (21, 22). As the tumor cells proliferate, they utilize a greater quantity of folate to sustain their growth. This is facilitated by the folate hydrolase activity of PSMA (23). While PSMA can be found on non-cancerous prostate tissue and other tissues, its high expression and high specificity for PCa cells make it a valuable biomarker for diagnosis, therapy, and monitoring treatment response (24). PSMA can be targeted with radioactive tracers for positron emission tomography/computed tomography (PET/CT) imaging, as well as targeted therapy with radioactive isotopes. This approach is known as theranostics, a tool that utilizes radiopharmaceuticals to both image and treat cancer by targeting PSMA (25). PSMA PET/CT demonstrates superior detection rates for recurrences and small metastases even in patients with low levels of PSA, such as Asian men (26). Given the unique molecular genotypes of PCa in Asian men, PSMA theranostics could offer a new direction for precise diagnosis and treatment. However, there is currently a lack of clinical reporting on the Asian population, and related explorations are urgently needed.

## 2 Mechanism of PSMA as a diagnostic and therapeutic target

PSMA exhibits higher expression levels in PCa cells, and these levels tend to increase with stage and grade of the tumor. This is particularly prominent in advanced PCa, metastatic disease, and when biochemical recurrence develops (BCR). This highly specific membrane surface expression feature makes it an extremely valuable diagnostic and therapeutic target (27). The structural distribution of the PSMA membrane provides surface accessibility for the molecules designed to interact with it, particularly the large extracellular domains and defined binding. This feature also allows for the development of diverse ligands, and high-affinity and specificity (28).

**Figure 2:**
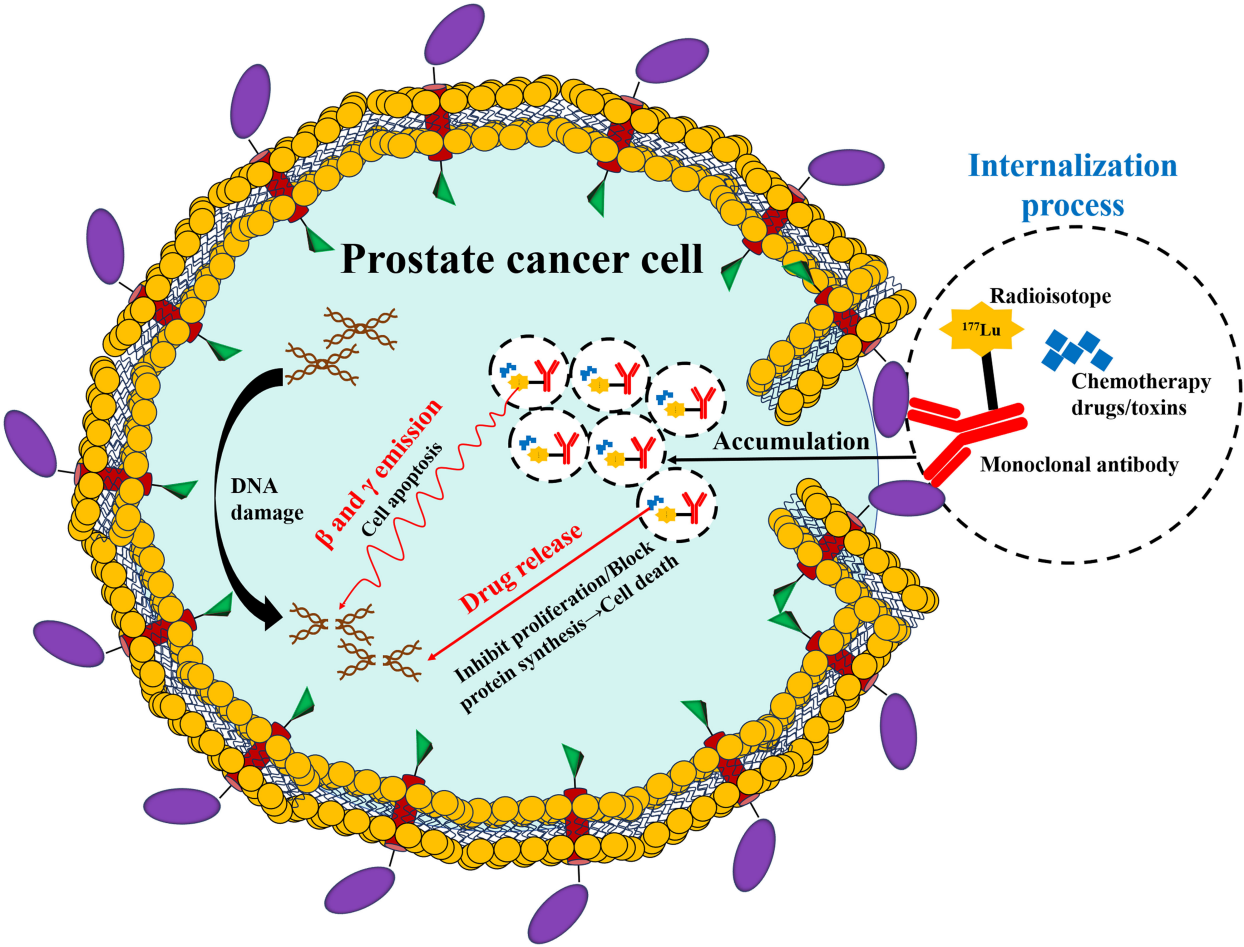
The diagnostic and therapeutic mechanisms of PCa using PSMA.

The core mechanism (**Figure 2**) of diagnosis and treatment based on PSMA lies in the utilization of the specific binding ability of the designed molecules that target PSMA (29). At the diagnostic level, these designed molecules are conjugated with radioactive tracers (such as a Ga-68, positron emitter), which attach to the cancer cells, marking them for precise localization through PSMA PET imaging, thus achieving visual tracking of the primary lesion and metastatic lesions (30, 31). At the therapeutic level, the molecules are labeled with radioactive molecules, which could include Lu-177 (a β-particle emitter) or Ac-255 (a α-particle emitter). Once attached to PSMA, these radioactive molecules decay and deliver radiation directly to the cancer cells [(PSMA-targeted radioligand therapy (RLT)] (32). This action induces DNA double-strand breaks, which trigger cancer cell death while minimizing damage to surrounding healthy tissues (33).

The currently developed molecules that target PSMA include small molecules (PSMA-617 and PSMA-I&T), monoclonal antibodies (J591 antibody and antibody-drug conjugates), and RNA aptamers (34, 35). Through design optimization, these molecules can be specifically designed to bind to the intracellular or extracellular domains of PSMA (36). **Table 1** provides a summary of the common clinical applications of PSMA-targeted ligands and radioactive molecules. With the advancements in clinical research, the development of PSMA-based theranostics has seen a shift from targeting the intracellular domain to targeting the extracellular domain, enabling internalization into viable cancer cells. This targets the limitations of earlier approaches that primarily targeted dying or necrotizing cells for the intracellular domain (56). PSMA-based theranostics has constructed a new diagnosis and treatment system integrating precise diagnosis and targeted therapy, providing a new solution for precise medical treatment of PCa, especially in the Asian population.

**Table 1:** PSMA drugs commonly used in clinical practice.

Ligands	Labels	Brief introduction	Clinical application	Year	Country	References
PSMA-617	^68^Ga	FDA approval, high accumulation in the urinary tract, high renal uptake, good pharmacokinetics, high targeted uptake efficiency	Diagnosis of primary lesions and lymph node metastases in PCa.	2023	China	(37)
	^177^Lu		Treatment of mCRPC.	2021 2023	India Pakistan	(38) (39)
	^225^Ac		A complementary agent to ^177^Lu-PSMA-617 or as part of its tandem therapeutic approaches.	2020	India	(40)
	^111^In		Detecting lymph node invasion during lymph node dissection in radical prostatectomy.	2018 2020	German	(41) (42)
PSMA-11	^68^Ga	FDA approval, high accumulation in the urinary tract	Detecting clinical recurrence after BCR following radical prostatectomy, and making adjunctive treatment decisions.	2019	Singapore	(26)
			Evaluating the therapeutic effect of ADT in PCa bone metastasis patients and detecting the stage and recurrence of PCa.	2021	Israel	(43)
			Identification of atypical metastasis of PCa (such as isolated parietal peritoneal metastasis).	2023	China	(44)
Piflufolastat (DCFPyL)	^18^F	FDA approval, low hepatic uptake, high accumulation in the urinary tract	Diagnosis of primary lesions and lymph node metastases in PCa, particularly in patients experiencing their first BCR.	2025	France, Belgium, Spain, Netherlands	(45) (46)
Flotufolastat (rhPSMA-7.3)	^18^F	FDA approval, good pharmacokinetics	Diagnosis of PCa, particularly for detecting recurrent PCa and monitoring salvage therapy.	2025	German	(47) (48)
rhPSMA-7	^18^F	Low accumulation in the urinary tract, low targeted uptake efficiency	Diagnosis of both primary and mPCa, less prevalent compared to ^18^F-DCFPyL and ^68^Ga-PSMA-11.	2024 2020	Austria German	(49) (50)
PSMA-1007	^18^F	Low accumulation in the urinary tract, high hepatic uptake	Diagnosis of PCa, including the detection of the primary lesion and the metastatic lesions.	2021	China	(51)
PSMA-I&T	^177^Lu	High accumulation in the urinary tract, high renal uptake, high targeted uptake efficiency	Treatment of mCRPC.	2022	China, Korea	(52)
	^68^Ga		Assessing tumor load in primary lesions of newly diagnosed prostate cancer, assisting in risk stratification, and predicting metastasis.	2022	China	(53)
PSMA-I&S	^99m^Tc	SPECT probe, the detection level is higher than PSMA-I&T and lower than PSMA-11	Preoperative SPECT/CT imaging and being adopted for PSMA-RGS in robot-assisted minimally invasive procedures.	2024	Italy Hungary	(54) (55)

ADT, androgen deprivation therapy; BCR, biochemical recurrence; PSMA, prostate-specific membrane antigen; FDA, Food and Drug Administration; PCa, prostate cancer; mPCa, metastatic prostate cancer; mHSPC, metastatic hormone-sensitive PCa; mCRPC, metastatic CRPC; RLT, radioligand therapy; SPECT, single-photon emission computed tomography; PSMA-RGS, prostate-specific membrane antigen radioguided surgery.

## 3 Application and value of PSMA imaging for diagnosing PCa

Elevated PSA levels can be caused by conditions other than PCa, such as benign prostatic hyperplasia (BPH) or prostatitis. This means a high PSA doesn’t always indicate cancer, thus causing a challenge to early and accurate diagnosis of PCa. Due to this low specificity, PSA testing can lead to unnecessary biopsies and treatments with potential side effects (57). Furthermore, PSA is not a structural or morphological feature, which means it cannot be directly used for PCa imaging (58). Therefore, the imaging typically relies on techniques such as CT, magnetic resonance imaging (MRI), and bone scans (BS), which can also have limitations when identifying subtle lesions. As a membrane-bound protein, PSMA can be targeted using radiolabeled molecules to visualize PCa cells. This means that PSMA imaging can often detect PCa that other imaging tests miss, particularly when PSA levels are low, thus providing more precise information (59). PSMA-based imaging tool, PSMA PET scan, utilizes a radioactive tracer that attaches to PSMA, allowing the scan to pinpoint the anatomic locations of the cells (60). It can also detect PCa metastasis to other parts of the body, as well as detect if the cancer treatment was effective. The effectiveness of the PSMA PET examination as a tool for the precise localization of tumor cells has been confirmed by studies showing a significant correlation between the level of PSMA uptake in the PSMA PET scan and the level of PSMA expression observed in the corresponding pathological sections.

**Figure 3:**
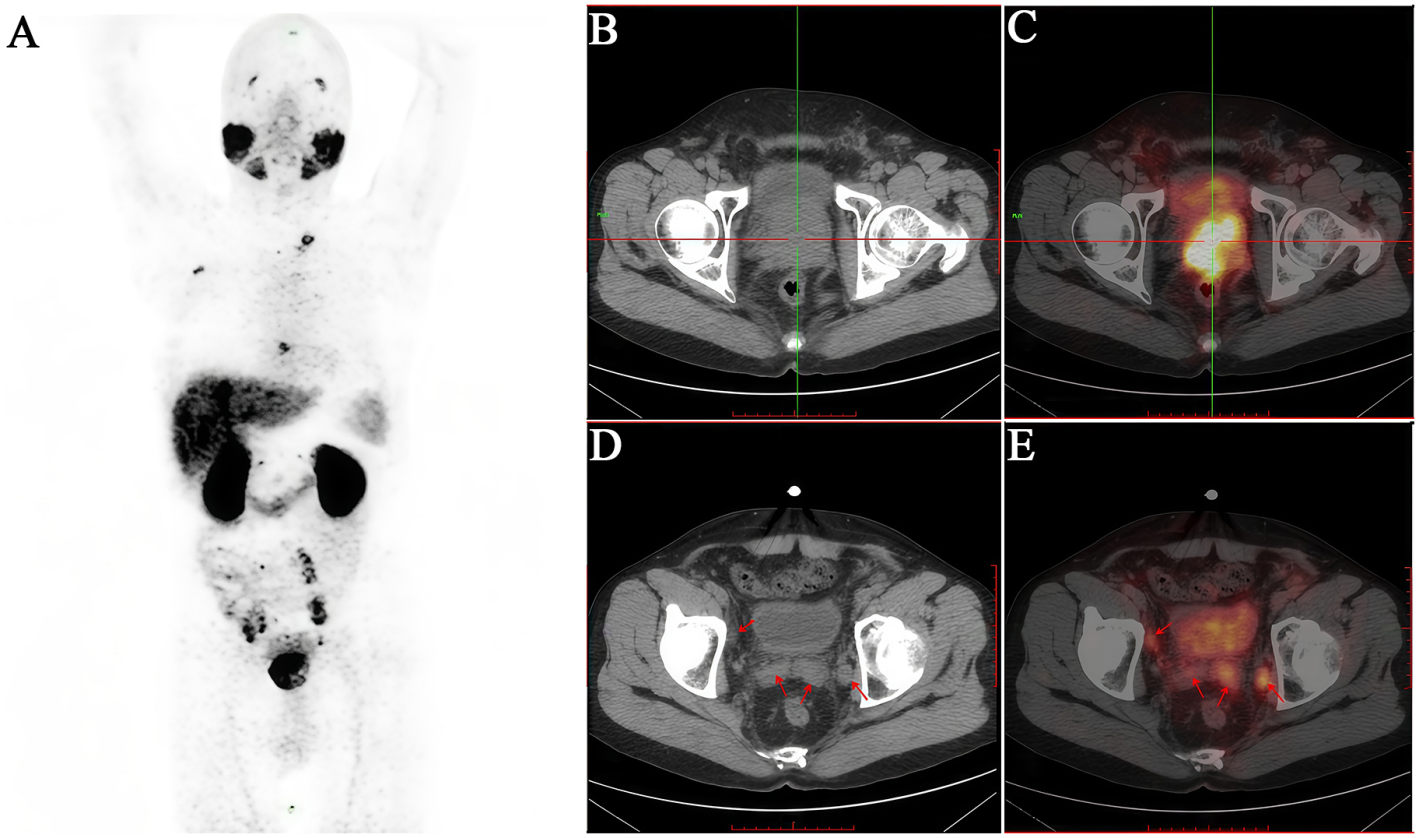
A 51-year-old male patient diagnosed with prostatic acinar adenocarcinoma (Gleason 4+4=8) by biopsy received an intravenous injection of ^18^F-PSMA. **(A)** PET/CT scan was performed 50 minutes later. **(B, C)** Multiple nodular and mass-like abnormal radioactive concentration shadows were observed in both lobes of the prostate (SUVmax 13.0). **(D, E)** The bilateral seminal vesicles were enlarged, especially the left side (SUVmax 3.0). They showed increased radioactive uptake. Additionally, the posterior wall of the bladder exhibited thickening with an indistinct boundary with the prostate, suggesting invasion. The patient subsequently underwent transurethral resection of bladder lesions, and the postoperative pathology was consistent with the results of the prostate biopsy.

**Figure 4:**
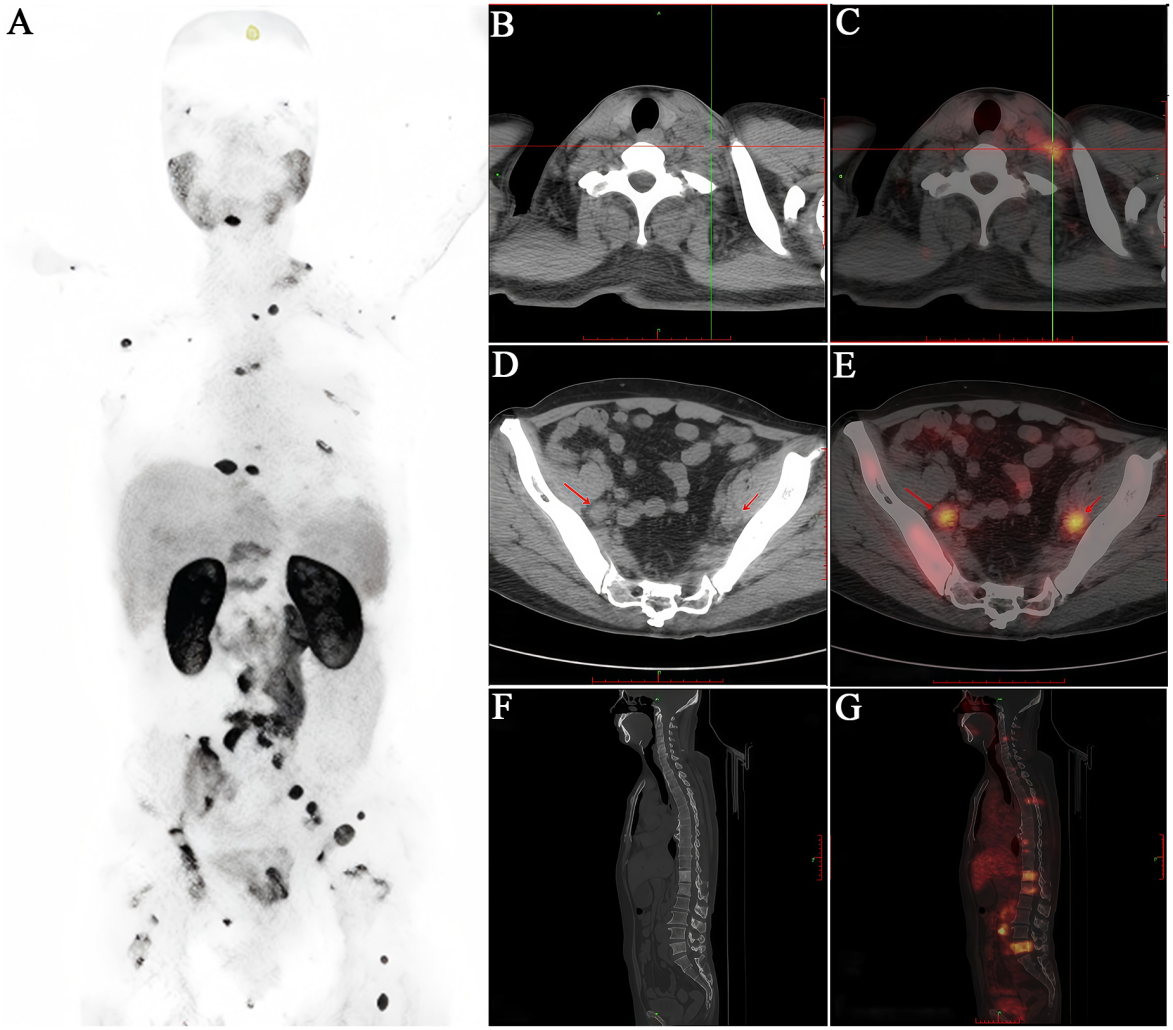
A 65-year-old male patient diagnosed with prostatic acinar adenocarcinoma (Gleason 4+4=8) by biopsy received an intravenous injection of ^18^F-PSMA. **(A)** PET/CT scan was performed 50 minutes later. **(B, C)** Multiple lymph nodes of varying sizes were observed in the left neck with increased radioactive uptake to different degrees (SUVmax 3.3). **(D, E)** Enlarged lymph nodes with increased radioactive uptake were seen around the bilateral iliac vessels (SUVmax 10.2). **(F, G)** Multiple vertebral bodies and some appendages of the spine, as well as the bones of the pelvis, showed significant bone destruction with increased radioactive uptake (SUVmax 6.7). It can be seen that the ^18^F-PSMA PET/CT scan comprehensively reflects the multiple metastatic burdens throughout the body of this PCa patient, providing a strong basis for clinical decision-making.

Based on the above background, PSMA-targeted molecular imaging, particularly PSMA PET/CT, is reshaping the diagnostic pattern of PCa with superb advantages. PSMA PET/CT is more accurate and sensitive at identifying local, regional, and distant metastatic disease (**Figures 3**, **4**), and this advantage is particularly significant in the diagnosis of BCR (61). Its enhanced sensitivity and specificity, particularly at PSA levels below 1 ng/ml, allow for early detection of recurrence and better guidance of treatment options (62). It is worth noting that PSMA PET/CT imaging uses two common types of PSMA tracers, namely Ga-68 and F-18. Between them, F-18 has a longer half-life than Ga-68, enabling it to offer slightly better spatial resolution (49). PSMA-based imaging can help in determining the extent of PCa, including lymph node involvement and distant metastases (63, 64). It is also highly sensitive in detecting BCR after treatment. These advantages can lead to more informed treatment decisions. Furthermore, and more importantly, it is less likely to produce inconclusive results compared to conventional imaging, and in some cases, may result in lower radiation exposure than the combination of CT and BS (65).

Following imaging, location, and staging of PCa facilitated by PSMA-based imaging, radioactive isotopes attached to a PSMA-targeted molecule are then delivered to the cancer cells, where they damage their DNA and lead to cell death. The PSMA-targeted molecules ensure that the radiation is delivered directly to the cancer cells, minimizing damage to the surrounding healthy tissues. The most commonly studied and used radionuclide to date is Lu-177. This radionuclide emits beta radiation, has a longer tissue range, and is used to treat smaller tumors and metastases, such as metastatic castration-resistant prostate cancer (mCRPC) (66). Ac-225 is also commonly used. It is an alpha-emitter; this radionuclide is more potent and has a higher therapeutic efficacy than Lu-177 due to its high linear energy transfer of the emitted alpha particles (67). This means that it induces double-stranded DNA breaks that are more difficult to repair. Both Lu-177 and Ac-225 hold significant promise for treating PCa in Asian men, particularly in advanced stages. In a prospective single-arm clinical trial, the application of ^177^Lu-PSMA-I&T RLT showed favorable responses in East Asian patients with mCRPC (52). The patients tolerated the treatment well and experienced tumor remission with significant PSA decline. Furthermore, the development of PSMA-based theranostics that specifically target bone metastases is also vital, as Asian men can be diagnosed with late-stage PCa that may involve metastasis (4). Ra-223 is a relatively new drug that functions as a bone-seeking calcium mimetic. Its structure is similar to calcium, allowing it to be taken up and incorporated into the bone matrix, particularly in areas of high bone turnover, as present in bone metastases. Ra-223 is an alpha emitter, which means it delivers potent and localized alpha radiation that effectively kills cancer cells while minimizing damage to healthy surrounding tissues (68).

Of course, in clinical applications, attention should also be paid to the heterogeneity of PSMA imaging results. The performance and application effects of different PSMA PET markers vary among patients at different stages and classifications, and it is necessary to optimize the selection in combination with individual characteristics (69). However, PSMA imaging technology is evolving into an essential core instrument for the diagnosis and management of PCa, offering the dual benefits of accurate visualization and tailored diagnosis, particularly providing a groundbreaking approach to the diagnosis of advanced PCa in Asian men.

## 4 PSMA-targeted therapeutic strategies and future perspectives of PCa

During the progression of PCa, most cases will ultimately advance to CRPC or mCRPC. As the final phase of PCa development, mCRPC is a significant contributor to mortality. Once the disease progresses to this phase, patients often have to rely on cytotoxic chemotherapy to prolong survival, but the prognosis is extremely poor. According to statistics, the 5-year overall survival rate of PCa patients in China (69.2%) is significantly lower than that in the United States (97.4%) (13). In addition to lifestyle, weak awareness of disease cognition, accessibility of screening programs and genomic differences, the proportion of advanced and new-onset metastases at diagnosis in Asian patients was significantly higher than that in the Western populations, making it difficult for traditional treatment strategies to meet the clinical needs of Asian PCa patients (7). Consequently, there is an urgent need for more effective new therapies to overcome these drawbacks.

Although the PSMA-targeted RLT has opened up new avenues for accurately targeting tumors by specifically recognizing the highly expressed PSMA on the surface of PCa cells, it does face certain limitations in clinical application. For example, the physiological overexpression of PSMA in salivary gland tissues leads to dose-limiting salivary gland injury during RLT (such as ^177^Lu-PSMA-617 and ^225^Ac-PSMA-617), which severely limits the intensity of treatment (70, 71). RLT has dose-limiting toxicity (such as myelosuppression), and some patients are unable to complete the entire course of treatment due to hematological toxicity (72, 73). Moreover, the absorption efficiency of radioactive drugs by tumors varies greatly, potentially resulting in inconsistent therapeutic outcomes (74). In light of this, a systematic organization of innovative treatment strategies targeting PSMA, along with a prospective exploration of future developmental directions, can assist in generating new concepts for enhancing the current targeted treatment approaches for Asian PCa patients.

### 4.1 Antibody-drug conjugate therapy

ADC therapy is a cutting-edge technology in tumor-targeted therapy. Similar to PSMA-targeted RLT, they both take advantage of the high expression characteristic of PSMA on PCa cells (75), but there are significant differences between the two. ADC utilizes monoclonal antibodies as targeted vectors, while RLT utilizes small-molecule ligands (76). Additionally, ADC utilizes cytotoxic chemotherapy drugs, which usually have a strong killing effect, while RLT exerts therapeutic effects by releasing radiation with radioactive isotopes (77). The monoclonal antibodies in ADC can precisely recognize and bind to PSMA. Once bound to PSMA, the ADC is internalized into the cancer cells through endocytosis. Inside the cell, ADC undergoes a series of intracellular processing, and their linkers break under the enzymatic cleavage or other mechanisms, releasing cytotoxic drugs (75). These drugs can disrupt key cellular processes, such as hindering microtubule polymerization, causing cell cycle arrest, and ultimately leading to the death of cancer cells. Clinical research data strongly confirm the anti-tumor activity of ADCs targeting PSMA. In the Phase II clinical trial of PSMA-MMAE, mCRPC patients treated with abiraterone/enzalutamide (abi/enz) showed positive changes in PSA levels, CTC status, and radiologic assessments (78). Among the patient group that did not receive chemotherapy, PSMA-MMAE also showed good potential (75). Up to 21% of the cases had a reduction in PSM of ≥50%, and 53% of the cases had CTC conversion. However, its overall response rate is still at a medium level. The main reason lies in the heterogeneity of PSMA expression on the surface of tumor cells, which makes it difficult for tumor cells to effectively take up ADC drugs. Furthermore, safety issues (such as neurotoxicity) (79) and treatment-related adverse events (80) are also key factors hindering the wide application of targeted ADCs for PSMA.

In the future, the development of PSMA-targeted ADCs necessitates further comprehensive investigation focus on improving therapeutic efficacy, reducing toxicity, and optimizing targeting mechanism (75). This goal can be achieved by optimizing dosages to minimize toxicity, refining the targeting mechanisms to enhance targeting precision (81, 82), as well as integrating technologies such as single-cell RNA sequencing (scRNA-seq) to screen patient subsets with high PSMA expression and therapeutic sensitivity, thereby facilitating the adoption of personalized therapy (83).

### 4.2 Cellular immunotherapy

Cellular immunotherapy, especially CAR-T cell therapy, is a rapidly evolving and significant area in PCa tumor treatment. This approach is gaining recognition due to its unique mechanism of action, where chimeric antigen receptors (CARs) (engineered protein receptors) enable T cells to specifically target and recognize tumor antigens like PSMA (84). Once CAR successfully binds to the antigen, T cells are immediately activated, thereby inducing apoptosis of cancer cells. Among numerous targets, PSMA is regarded as one of the most reliable targets for CAR-T cell immunotherapy. The results of relevant basic experiments and Phase I clinical trials show that CAR-T cell immunotherapy has demonstrated certain therapeutic effects on PCa (85–90). However, the current research is still in the preclinical phase, and further in-depth exploration is needed before it can be widely applied in clinical practice (91–93). While CAR-T immunotherapy shows impressive applicability, it may present with toxic side effects such as cytokine release syndrome (94). Bispecific T-cell engager (BiTE) immunotherapy is another breakthrough in the field of cellular immunotherapy. It has been approved for the treatment of certain cancers. BiTE is essentially a special ligand composed of two different antibodies with single-chain variable fragment domains. Its mechanism of action involves combining CD3 molecules and the surface antigens of tumor cells to activate the T cells of patients, enabling them to precisely eliminate tumor cells. This process circumvents the co-stimulation model or major histocompatibility complex mechanisms, thereby leading to enhanced efficacy (95, 96).

Compared to RLT, cell immunotherapy focuses on activating or modifying the patient’s immune cells to recognize and attack cancer cells. Its key advantage is the potential for long-lasting immunity and a durable response by training the immune system to remember and target cancer cells. Future research should be dedicated to optimizing CAR design and enhancing the recognition and killing ability of T cells against tumors. For instance, the Echo-Back-CAR-T cells developed by Liu et al. (97) integrate a heat-sensitive promoter with the CAR signaling circuit aimed at targeting PSMA, resulting in prolonged tumor suppression with reduced side effects. This paves the way for CAR-T cell immunotherapy in the realm of solid tumors like PCa. Additionally, the combination of this treatment with immune checkpoint inhibitors (such as anti-PD-1 antibodies) could be employed to enhance anti-tumor immune responses through PSMA-mediated immune cell recruitment (83, 98). This approach may recruit immune cells to the tumor site, potentially reversing the tumor’s ability to evade the immune system.

### 4.3 Other PSMA-targeted therapies

Photodynamic therapy (PDT) and photothermal therapy (PTT) are minimally invasive treatment methods for PCa cells that utilize PSMA as the molecular target and achieve precise intervention in tumor cells through molecular targeting strategies. DT delivers photosensitizers to tumor cells by relying on the specific binding of PSMA-targeted ligands to PSMA receptors on the surface of cancer cells. In tumor cells expressing PSMA, photosensitizers are effectively enriched through specific binding (99). When exposed to a specific wavelength, the excited photosensitizers transfer energy to the surrounding oxygen molecules, generating highly reactive oxygen species (ROS), thereby leading to apoptosis or necrosis of cells. PTT exerts its function by taking advantage of the photothermal conversion characteristics of photothermal agents (PTAs). After coupling the PSMA-targeted agent with photothermal materials, the resulting PSMA-targeted photothermal agent can be specifically enriched in tumor tissues (100). When near-infrared (NIR) light is used to irradiate the tumor site, the photothermal agent can efficiently absorb the NIR light energy and convert it into thermal energy. The localized heat generation increases the temperature within tumor cells, leading to protein denaturation and cell membrane destruction. It is worth noting that, compared with PDT, which relies on oxygen to function, PTT has more advantages in the treatment of hypoxic tumors because its mechanism of action does not depend on oxygen participation and can effectively overcome the limitations of the hypoxic microenvironment of tumor tissues on therapeutic effects. Compared with RLT, PDT and PTT do not require the use of radioactive substances, significantly reducing the risk of radiation-related adverse reactions, which is of great significance in improving the safety of treatment. However, at present, these two treatment methods are still in the preclinical research and clinical trial stage. There is still considerable room for development in aspects such as the optimal design of photosensitizers/photothermal agents, the improvement of targeted delivery efficiency, the precise regulation of treatment parameters, and the exploration of combined treatment strategies. In the future, with continuous technological breakthroughs, PDT and PTT are expected to become important means in the field of precise treatment for PCa, bringing new treatment options and survival hope to patients.

PSMA radioguided therapy can also include surgery. This procedure, known as PSMA radioguided surgery (PSMA-RGS), is a surgical technique that utilizes gamma probe imaging guided by PSMA ligands to facilitate intraoperative tumor resection, particularly PCa (101, 102). PSMA-RGS allows the precise pinpointing and removal of PSMA-positive cancerous tissue. The radioactive isotopes (like Lu-177 and Ac-225) accumulate at the sites with high PSMA. During surgery, a gamma probe hand held by a surgeon, detects the gamma radiation. This is used to measure the amount of radiation emitted from the surgical site, allowing the surgeon to differentiate between the tumor site (areas with high PSMA expression) and surrounding healthy tissue (103). This method is especially vital in cases of BCR after initial treatment or in primary prostatectomy to improve lymph node dissection and reduce the risk of positive surgical margins (104). In 2015, the Technical University of Munich carried out the first successful utilization of ^111^In-PSMA-I&T RGS (^111^In-PSMA-RGS) in patient treatment, thereby showing its high value for intra-operative detection of even small metastatic lesions in patients with PCa (105). In a study conducted in 2015 by Maurer et al. (106), the effectiveness of PSMA radio-guided surgery for detecting metastatic lymph nodes was confirmed using ^68^Ga-labeled PSMA. Metastatic lymph nodes smaller than 1 cm were noted, and additional lesions adjacent to the known tumor lesion that had not been visualized on preoperative ^68^Ga-PSMA-HBED-CC PET imaging were identified during PSMA-RGS in two patients. Comparable to medical targeted therapies, PSMA-RGS allows for the precise identification of PSMA-expressing cells during surgery. In a retrospective study conducted by Rauscher et al. (107), 31 patients with localized recurrent PCa who underwent salvage surgery were included. PSMA-RGS was performed using an ^111^In-labelled PSMA ligand. The study results demonstrated that the sensitivity, specificity, and accuracy of ^111^In-PSMA-RGS were 92.3%, 93.5%, and 93.1%, respectively. Schilham et al. (108) conducted a prospective study involving 20 patients with newly diagnosed PCa to evaluate the safety and effectiveness of ^111^In-PSMA RGS. The results showed a successful removal rate of 88% (43 out of 49) for lesions identified by ^18^F-PSMA PET. The utilization of ^111^In-PSMA RGS enabled the identification and resection of 59% (29 out of 49) of the targeted lesions, with lymph node metastases detected in 97% (28 out of 29) of cases. However, there were an additional 29% (14 out of 49) of resected lymph nodes that were not detected using the same technique, two of which contained metastases. Overall, it was found that the procedure of ^111^In-PSMA RGS was a safe and feasible clinical procedure. It should be noted that with its incredible success, the radioactive exposure from the radioactive drugs used in RGS is concerning for the medical staff. Consequently, safer approaches have been underway. Radiation-free PSMA-targeted fluorescence-guided surgery (PSMA-FGS) is a safer option in development; in fact, it has advanced from preclinical studies to clinical trials. Professor Hamdy from the University of Oxford developed and synthesized the PSMA-targeted fluorescent probe IR800-IAB2M in 2024. It was tested on 23 patients in a human study of RARP (ISCRCTN10046036). The overall sensitivity and specificity for detecting non-lymph-node extra-prostatic cancer tissue were 100% and 65%, respectively. Furthermore, the sensitivity and specificity for detecting positive lymph nodes were both 64%, indicating that the use of intraoperative imaging with IR800-IAB2M for PCa tissue is feasible and safe (109).

## 5 Conclusions and prospects

In the past decade, PSMA has emerged as a focal point in PCa research. As PSMA ligand technology continues to advance and mature, an increasing number of PSMA ligands are being utilized in clinical trials. Extensive research has demonstrated that PSMA imaging technology is capable of detecting lymph node metastases, bone metastases, and distant metastatic lesions that are challenging to identify using conventional imaging methods. This capability holds significant clinical implications for treatment planning and follow-up monitoring in patients with advanced PCa (110, 111). PSMA PET/CT is advancing at an unprecedented pace, marked by a substantial increase in the number of published studies and clinical trials. In accordance with the guidelines from the European Association of Urology (EAU), PSMA PET/CT is recommended for evaluating the likelihood of PCa recurrence and distant metastasis in patients with PSA levels exceeding 0.2 ng/ml who are candidates for salvage therapy. PSMA PET/CT is capable of accurately evaluating the size of the lesion and its relationship with surrounding tissues, thereby facilitating more precise tumor-nodes-metastasis (TNM) staging for PCa. This modality provides critical guidance for targeted biopsies, detection of BCR and metastasis, supports clinical decision-making processes, and enables effective evaluation of therapeutic efficacy (112, 113). However, it is important to highlight that the use of PET/CT in conjunction with PSMA imaging may not be appropriate for all patients with PCa. A comprehensive evaluation of the patient’s clinical stage, prior diagnostic and therapeutic history, as well as other pertinent factors, is essential. Future research should focus on thoroughly characterizing the patient population and clearly delineating the distinct features and requirements associated with different clinical stages. Moreover, domestic and international studies have yet to reach a consensus regarding the tangible benefits of relying solely on PSMA PET/CT for decision-making in PCa diagnosis and treatment (114, 115). Consequently, there is an urgent requirement for additional multicenter, randomized, controlled, and prospective studies to strengthen the scientific rigor and credibility of the findings.

PSMA-targeted therapy, particularly using RLT like ^177^Lu-PSMA-617, has shown significant clinical benefits in reducing PSA levels and extending survival in patients with PCa. Nevertheless, clinicians must rigorously assess patient eligibility before initiating treatment to prevent unnecessary interventions. This assessment is vital because PSMA-targeted therapy, particularly PSMA-RLT, delivers radiation directly to PSMA-expressing cancer cells and therefore requires a high level of PSMA expression on the tumor to be effective. This necessitates the development of individualized treatment plans. Molecular profiling (such as gene mutations, aberrant signaling pathways), including genomics, transcriptomics, and proteomics, holds significant promise for refining patient selection and optimizing treatment efficacy in PSMA-targeted therapies for PCa (98). Additionally, dynamic treatment adjustments based on real-time PSMA molecular imaging (such as modifying dosages or combining with chemotherapy/immunotherapy based on changes in PSMA expression) will enable “real-time intervention”. Furthermore, additional in-depth investigations are required to assess the potential risk of damage to normal tissues resulting from high-dose PSMA drugs.

Key directions in mechanistic research related to PSMA’s role in the tumor microenvironment include investigating its involvement in angiogenesis and shaping immunosuppressive niches, which can inform combination therapies with anti-angiogenic or immunomodulatory agents (98). By understanding the diverse mechanisms of resistance including PSMA gene downregulation or compensatory activation of pathways (like androgen receptor reactivation) and developing targeted strategies, particularly by using combination therapies (integration of androgen receptor antagonists), it is hoped that long-term outcomes for patients with advanced PCa can be significantly improved.

PDT and PTT have demonstrated substantial efficacy in preclinical studies and have been successfully integrated with chemotherapy and other technologies. Nevertheless, the majority of these researches remain at the preclinical stage, and there is an urgent need to accelerate their clinical translation. In addition, the PSMA-RGS technology can be employed for PSMA ligand-guided precision surgery. However, the radiation effects on both the operator and the patient must be comprehensively evaluated. In this regard, PSMA-FGS serves as an effective and feasible alternative solution. In recent years, the rapid advancement of near-infrared fluorescence second window (NIR-II, wavelength range 900–1700 nm) technology has enabled significant progress in NIR-II fluorescence imaging for PCa marker targets and its guidance applications in surgical procedures. This approach demonstrates superior advantages in terms of signal-to-noise ratio, real-time imaging, multi-modality, and application scope compared to traditional optical imaging and near-infrared fluorescence first window (NIR-I) imaging. These enhancements provide a promising foundation for achieving complete tumor resection during surgery (109, 116–118). Therefore, PSMA-FGS offers a novel research avenue for the precision therapy of PCa and demonstrates significant potential for clinical translation.

In summary, the theranostic strategy targeting PSMA is anticipated to become one of the standard approaches for the routine screening and management of PCa in the future (**Figures 3**, **4**).
